# Low dose hydrophilic statins are the preferred agents for females at risk of osteoporosis

**DOI:** 10.1016/j.bonr.2021.101152

**Published:** 2021-11-30

**Authors:** Alisa Antonenko, Aoife Leahy, Mihaly Babenko, Declan Lyons

**Affiliations:** University Hospital Limerick, St. Nessan's Road, Dooradoyle, co. Limerick V94F858, Ireland

**Keywords:** Osteoporosis, Statins, Atherosclerosis, Bone mineral density, Dose dependent

## Abstract

**Objectives:**

The correlation between atherosclerosis and osteoporosis, independent of age, is clear. Multifactorial co-dependence between bone mineral density (BMD) and statin dose has been proposed. It is hypothesised that inhibition of the synthesis of cholesterol will also inhibit the synthesis of sex hormones and Vitamin D, negatively affecting BMD. This study aims to evaluate hydrophilic and non-hydrophilic statins effect on osteoporosis and analyse any possible superiority of one agent over the other within the group.

**Methods:**

We identified 538 caucasian females who had a DEXA scan performed between 2002 and 2016 (age 60–89) in one DEXA center in Mid-West Ireland. A DEXA T-score results were analysed in the current study. Two hundred fifty females were not on statin therapy, and 323 females were on statin therapy. Females on therapy were separated into the atorvastatin group (*N* = 190), rosuvastatin group (*N* = 97), and pravastatin group (*N* = 36), comprising low dose and high dose groups. All anonymised data were analysed with SPSS statistical. To test the hypothesis that lower bone density is associated with high dose statins, an independent sample *t*-test was performed. The one-way between-groups ANOVA test was used to test the hypothesis that the BMD level depended on the statin's potency.

**Results:**

Statin-naïve females have a statistically higher bone mineral density in the lumbar spine, t (538) = 3.42, *p* < 0.05 and in hip t (538) = 4.99, p < 0.05 than females on statin therapy. There was a significant difference in patient's age between the group, and no significant correlation was found between the patient's age and type of statin or bone density. In the atorvastatin group statistically, significant results were obtained both for spine and hip bone mineral density, t (188) = −5.61, *p* < 0.05 and t (188) = −3.62, p < 0.05, respectively. In the rosuvastatin group, statistically, a significant result was noted for bone mineral density of hip t (95) = −3.52, *p* < 0.05. This demonstrates a dose-dependency between bone mineral density and the dose of the statin. The independent between-group ANOVA yielded a statistically significant effect, F (2, 59) = 6.69, p < 0.05, η2 = 0.21 in the spine. Thus, patients on lipophilic statins had statistically lower BMD than females on hydrophilic statins. Multilinear regression analysis identified that age is not a statistically significant contributor in our analysis; however, the trend of decrease in bone mineral density with women's age is acknowledged by authors.

**Conclusions:**

The study results support the theory that bone mineral density decreases with an increase in a statin dose, and hydrophilic statins, like pravastatin, have a better metabolic profile in the lumbar spine than lipophilic agents.

## Highlights

• Elderly females on statin therapy has lower bone mineral density in comparison to statin-naive females.
• Females on high dose atorvastatin in comparison to females on the low dose had statistically lower bone mineral density.
• Women in rosuvastin group had lower bone mineral density in the hip in comparison to females on low dose therapy.
• Hydrophilic statins in high doses are superior in terms of supporting bone health among females on statin therapy.

## 1 Introduction

Osteoporosis and cardiovascular disease are both serious public health concerns, as their prevalence increases with age. It is estimated that by 2050, hip fractures in women will increase by 240%, and in men, by 310% compared to the year 1990. There is a combined lifetime risk of forearm, vertebral and hip fracture of 40%, which correlates with the risk of cardiovascular events (International osteoporosis foundation, 2017).

Recent studies have suggested a clear correlation between atherosclerosis and osteoporosis, independent of age. Yamauchi M. et al. in 2015 showed a direct negative relationship between aortic calcification and femoral neck bone mineral density in an older woman after adjustment for age (Yamauchi et al., 2015). Zhang Y. states that patients with lower BMD are inclined to more severe coronary artery lesions (Zhang et al., 2020). Furthermore, Makovey J. 2009, in his study illustrates a modest inverse relationship between the lumbar spine and whole-body BMD and serum triglycerides and LDL levels in postmenopausal women and HDL in pre-menopausal women (Makovey et al., 2009). Extensive research has proven the positive impact of statin therapy on the cardiovascular system. This motivated researchers to investigate whether HMG-CoA reductase inhibition, the primary mechanism of statins, affects bone mineral density. Chung et al., reports 2.9% increase in bone mineral density among type 2 diabetes mellitus patient on cholesterol-lowering therapy (Chung et al., 2000). Other studies show no effect of statin therapy on bone mineral density or fracture risk among patients (LaCroix et al., 2003; Seeger et al., 2002; Bone et al., 2007). Rejnmark and colleagues hypothesise that differences in findings are secondary to the study design used (mainly non-randomised studies) and indication for statin therapy to be hypercholesterolaemia disorder, in opposition to performing randomised controlled trials among “healthy drug users” (Rejnmark et al., 2002).

Furthermore, evidence shows a difference in bone cell penetration in patients on different statin groups (lipophilic and hydrophilic statin groups) (Skripnikova et al., 2019; Rejnmark et al., 2006; Shahrezaee et al., 2018).

There is no official recommendation for using statin therapy in patients with osteoporosis as the data is very heterogeneous regarding dose-effect correlation, statin effect on sex hormones levels (estradiol and testosterone), and vitamin D levels (Leutner et al., 2019; Thabit et al., 2014; Hsia et al., 2002; Varri et al., 2016; Orces et al., 2020).

Despite significant statin-induced improvement in endothelial function and decreases in circulating pro-inflammatory markers, previous reports indicated that by reducing cholesterol serum concentrations, the capacity of steroidogenic tissues to produce adrenocortical hormones and sex steroids (including testosterone and estrogens) decrease (Fragasso et al., 2019). Additionally, the magnitude of the decrease in testosterone has been shown to be directly proportional to the dosage of statin therapy (Keyser et al., 2015). Since statin main mechanism is to inhibit the synthesis of cholesterol, it is hypothesised that it will also inhibit the synthesis of vitamin D (cholesterol is a precursor of Vitamin D). Another mechanism has been proposed because both 25(OH) vitamin D and statins are metabolised in the liver by a common enzyme of the cytochrome P450 system called CYP3A4. Occupation of the active site of this enzyme by statins may lead to a change in serum 25(OH) vitamin D levels. However, data are diverse at this time, and there is no common conclusion on this matter (Yamauchi et al., 2015; Mazidi et al., 2017).

In the current study, we investigated the relationship between the dose of statins and bone mineral density. We also explored possible differences within the class of statins and demonstrated a discrepancy in BMD among woman on statin therapy and statin therapy free.

## 2 Materials and methods

### 2.1 Study design

A retrospective cohort study was performed among woman 60–89 years of age, in the one DEXA center (one regional hospital) in the Mid-West Region of Ireland. The aim of the study is to evaluate effect of hydrophilic and non-hydrophilic statins on osteoporosis and to analyse any possible superiority of one agent over the other within the group.

### 2.2 Study population

Among 3381 records available, 1675 (49.5%) were assigned to the female population, who attended DEXA center for the first time. 1079 females had a complete prescription and questionnaire attached to the DEXA results, aged 30–89 years of age. Since authors were interested in analysing the influence of statin therapy on bone mineral density among older woman, 538 reports had complete prescriptions and questionnaires available, and patients were over 60 years of age (49.7% of the elderly female population with a complete record). Among them, 323 women (60%) were on statin therapy.

### 2.3 Data sources

Only anonymised DEXA results from 2002 until 2016 with medication prescriptions at the time of the test being done and standard questionnaires attached to them were available to the authors. This limited the study, as no further information could have been requested (complete list of comorbidities, a full history of exposure to osteoporosis treatment).

A DEXA T-score results were analysed in the current study. T-scores were assessed in two categories: hip T score and lumbar spine T score.

Females on statin therapy were separated into three groups depending on the type of agent they were on, and each group was also divided into high and low dose statin. In the atorvastatin group low dose considered 10 mg, 20 mg; high dose – 40 mg, 80 mg. Rosuvastatin low dose group 5 mg, 10 mg; and high dose group – 20 mg, 40 mg. The Pravastatin group was divided into low and high dose groups 10 mg, 20 mg and 30 mg, 40 mg, respectively.

### 2.4 Exclusion criteria

Exclusion criteria: long term steroid use (oral/inhalers/nebuliser forms), hormonal replacement therapy or bisphosphonate treatment – from available prescription; previous or active cancer history, known genetic conditions that have an impact on bone or cardio-vascular system – information from the questionnaire.

### 2.5 Statistical analysis

All data were analysed with SPSS statistical software. Patients mean age was calculated for each group separately and can be found in Table 1. The distribution of the women on different types of statins was analysed with crosstabulation and chi-square test between patient's age and type of a statin therapy calculated to establish associations between age and type of statin.

**Table 1:** Patient's mean age by group.

Patients' group	Atorvastatin low dose	Atorvastatin high dose	Rosuvastatin low dos	Rosuvastatin high dose	Pravastatin low dose	Pravastatin high dose
Mean age (Standard deviation (SD))	73.97 (SD9.36)	75.53 (SD7.97)	75.74 (SD7.69)	73.48 (SD7.74)	73.41 (SD7.35)	73.83 (SD8.13)

Pearson's correlation between females' age and a DEXA T-score (spine and hip joint separately) was performed using bivariate correlation to establish an association.

To test the hypothesis that lower bone density is associated with high dose statins, an independent sample *t*-test was performed. As shown in Table 2, the distributions in all groups were standard for conducting t-test (i.e., skew <│2.0│ and kurtosis < │9.0│). Additionally, the assumption of homogeneity of variances was tested and satisfied via Levene's F test (Table 3).

**Table 2:**
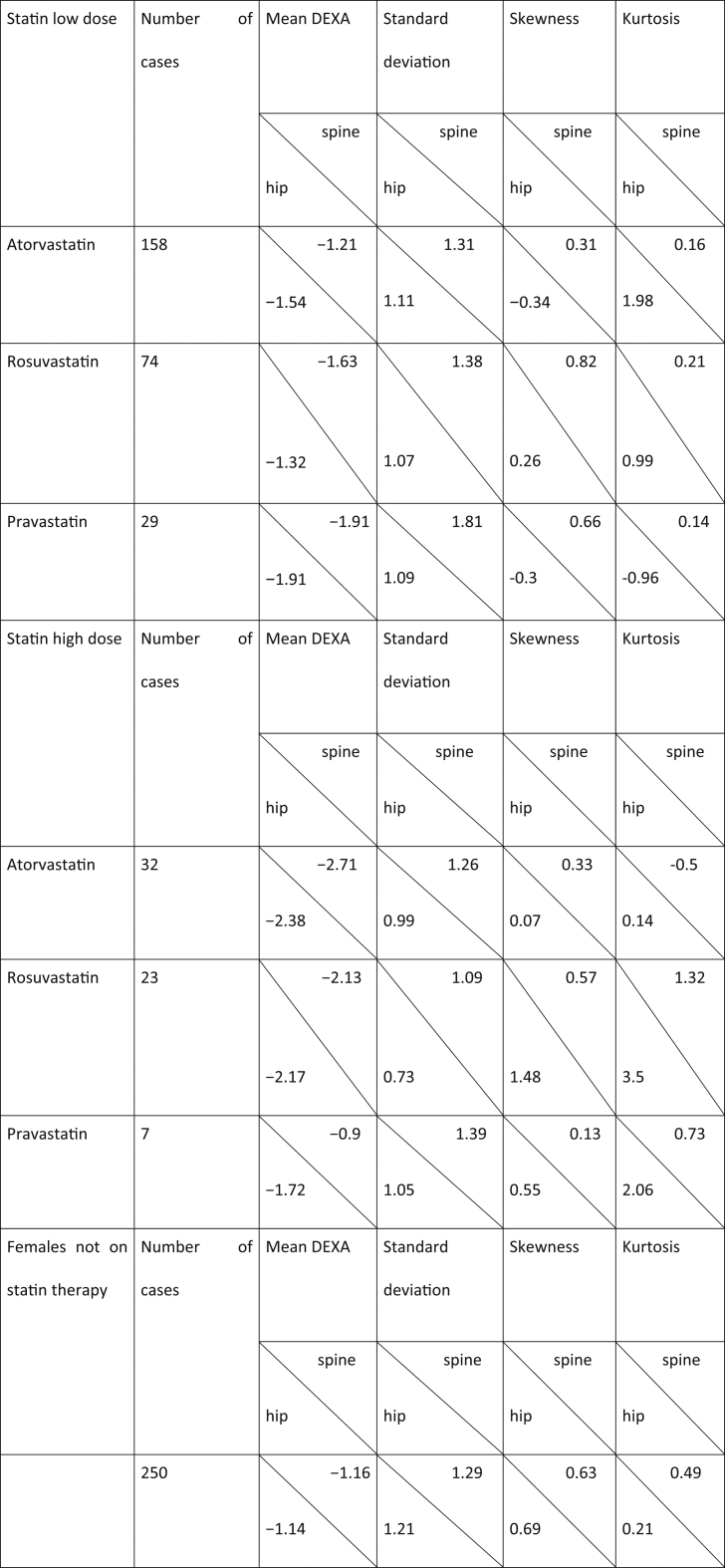
Descriptive statistics associated with low bone mineral density.

**Table 3:**
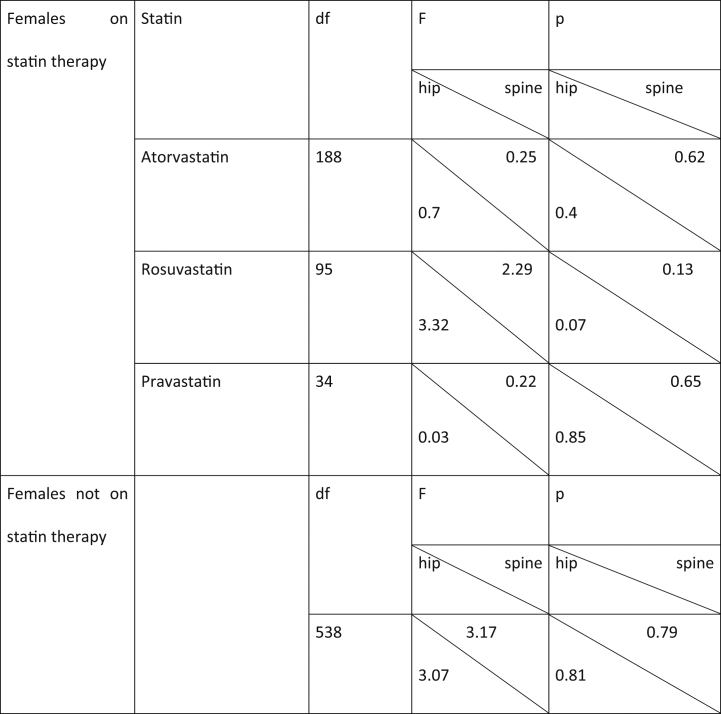
Levene's test for assumption of homogeneity.

Moreover, a one-way between-groups ANOVA test was used to test the hypothesis that the level of BMD also will depend on the potency of a statin. Patients were assigned categorical variables depending on the type and dose of statin they received. The assumption of homogeneity of variances was tested and satisfied via Welch and Brown –Forsythe test.

The hypothesis that changes in bone mineral density is secondary to the contribution of age as a co-factor was tested using multilinear regression analysis, backward method. To perform this analysis, patients were separated into three groups by age: 60–70 y.o.-group 1, 71–80 y.o. – group 2, 81–89 y.o. – group 3.

### 2.6 Ethical approval

The current study received ethical approval, AON approval and was approved by a clinical site (REC Ref 084/2020).

## 3 Results

### 3.1 Participants characteristics

We identified 538 caucasian females who had a DEXA scan performed in 2002–2016. 250 females were not on statin therapy, and 323 females were on one of three statins.

Atorvastatin group (*N* = 190), Rosuvastatin group (*N* = 97), and Pravastatin group (*N* = 36) comprised of low dose and high dose groups.

The summary of distributions of females on different types of statin therapy presented in Table 4, the result of the Chi-Square test association show there is no significant association between type of statin patients are on and their age (c2(df10, N323) =3.78, *p* = 0.957). A Pearson's r data analysis revealed a weak non-significant negative correlation between age and DEXA lumbar spine T-score *r* = −0.12 (*p* = 0.31), and moderate non-significant correlation between age and DEXA hip T-score, *r* = −0.34 (*p* = 0.538).

**Table 4:** The summary of distributions of females on different types of statin therapy.

Statin group	Age group			
	1 (60–70 y.o.)	2 (71–80 y.o.)	3 (81–89 y.o.)	Total
Atorvastatin low dose	53a	66a	39a	158
Atorvastatin high dose	9a	13a	10a	32
Rosuvastatin low dose	22a	28a	24a	74
Rosuvastatin high dose	8a	11a	4a	23
Pravastatin low dose	11a	12a	6a	29
Pravastatin high dose	2a	3a	2a	7
Total	105	133	85	323

Each subscript letter denotes a subset of age group categories whose column proportions do not differ significantly from each other at the 0.05 level.

### 3.2 Modeling

The independent samples *t*-tests were associated with statistically significant results in the relationship between the increase of the statin dose and bone density. Females not on statin treatment had statistically higher bone mineral density in the lumbar spine, t (538) = 3.42, *p* < 0.05 [Mean DEXA T score −1.16 SD ± 1.29] and in the hip t (538) = 4.99, p < 0.05 [Mean DEXA T score −1.1 SD ± 1.21] than females on statin therapy [Mean DEXA T score −1.59 SD ± 1.4 in the spine and −1.65 SD ± 1.10 in the hip respectively].

Statistically significant results were obtained when the comparison between low dose and high dose groups was performed. Mean DEXA T score spine in low dose atorvastatin group measured 1.21 SD ± 1.31 vs −2.71 SD ± 1.26 in high dose atorvastatin group (t (188) = −5.61, *p* < 0.05) and in the mean hip.

**Fig. 1:**
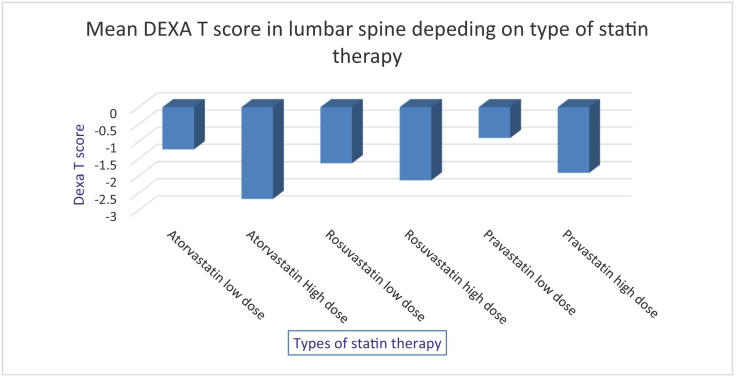
Mean DEXA T score mineral density in lumbar spine depending on dose and type of statin in females' 60–89 y.o.

**Fig. 2:**
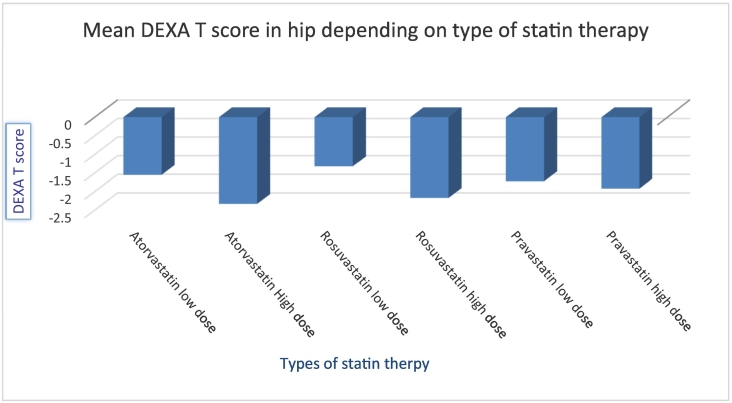
Mean bone mineral density in hip depending on dose and type of statin in females' 60–89 y.o.

T score in low dose atorvastatin group was −1.54 SD ± 1.11 vs in high dose group −2.38 SD ± 0.99 (t (188) = −3.62, p < 0.05). In the rosuvastatin group, a statistically significant result was noted in the hip, with a mean T DEXA T score −1.32 SD ± 1.07 in a low dose group vs −2.12 SD ± 0.73 in a high rosuvastatin dose group (t (95) = −3.52, p < 0.05). No statistically significant difference in the pravastatin group was found. Fig. 1, Fig. 2 represent a type of statin therapy, dose and respective mean T-score in the lumbar spine and hip joint, respectively.

Thus, females on higher doses of atorvastatin had significantly lower BMD both in the spine and the hip. Females in the rosuvastatin group had lower BMD in the hip compared to females on low dose therapy. Also, the effect size was measured with the help of Hedges' g (sample sizes are different in numbers), and results are recorded in Table 5.

**Table 5:** Hedges' g of estimation effect size.

Non statin to statin therapy			
	0.44	0.31	Low
Statin (high to low dose)	DEXA spine	DEXA hip	Effect
Atorvastatin	1.1	0.71	High
Rosuvastatin	0.39	0.85	Median/High
Pravastatin	0.58	0.18	Median/Low

**Fig. 3:**
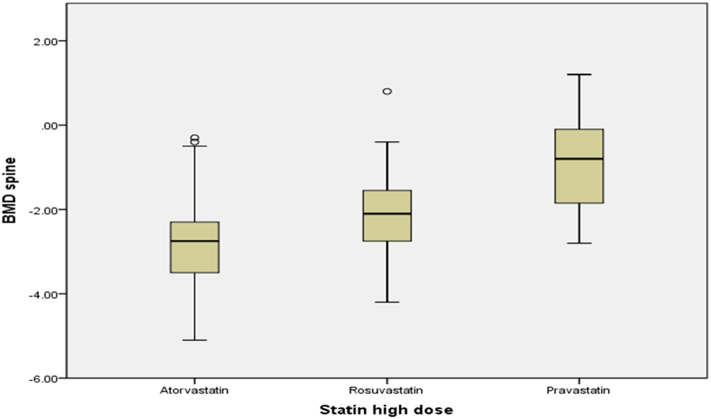
Bone mineral density presented by a DEXA T score in spine in females on high dose statins bar graph.

Furthermore, one – way ANOVA test was used to test the hypothesis that the level of BMD also depended on the potency of a statin. The assumption of homogeneity of variances was tested and satisfied via Welch and Brown –Forsythe test, W (2) =5.33, *p* < 0.05 and BF (2) =6.14, *p* < 0.05. The independent between-group ANOVA yielded a statistically significant effect, F (2, 59) = 6.69, p < 0.05, ⴄ2 = 0.21 in the spine. Thus, patients on lipophilic statins had statistically lower BMD than females on hydrophilic statins. The visual depiction of the means and 95% confidence intervals is presented in Fig. 3. No statistically significant difference in BMD of the hip was found. There was no statistical difference in BMD in female patients who were taking low dose statins.

**Fig. 4:**
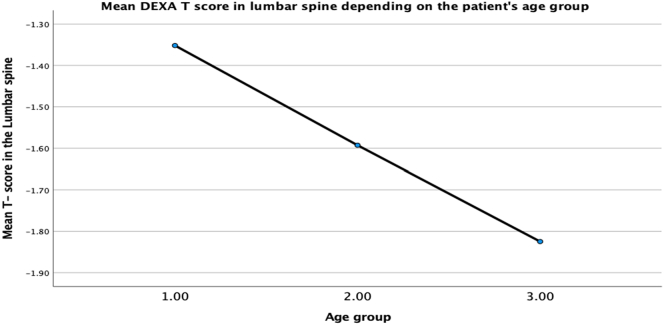
Reduction in Mean T-score in the Lumbar spine through age groups of female patients (60–70 y.o.-group 1, 71–80 y.o. – group 2, 81–89 y.o. – group 3).

**Fig. 5:**
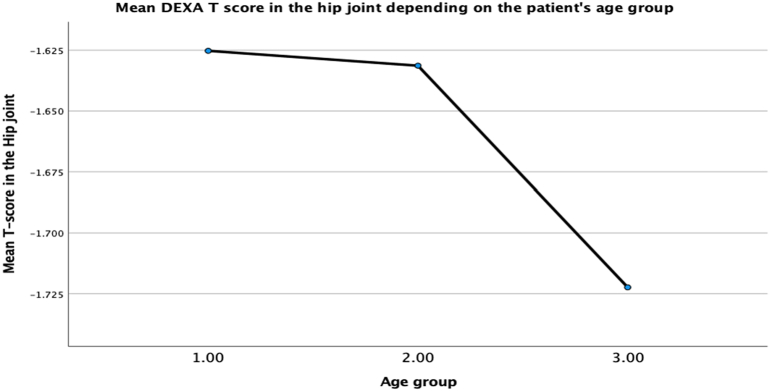
Reduction in Mean T-score in the hip through age groups of female patient's age (60–70 y.o.-group 1, 71–80 y.o. – group 2, 81–89 y.o. – group 3).

The hypothesis that changes in bone mineral density is secondary to the contribution of age as a co-factor was tested using multilinear regression analysis, backward method. Results have shown that 13% of the DEXA lumbar spine T score variance can be accounted for by the three contributors, collectively, F (5,317) =9,12, *p* < 0.001. Looking at the unique individual contributions of the predictors, the results show that high atorvastatin dose (b = −0.315, *t* = −5.77, *p* < *0*.*001*), high rosuvastatin dose (b = −0.172, *t* *=* *−0*.*318*, *p* *=* *0*.*02*) negatively predict bone mineral density. The report also revealed that female patients in group 3 (80–89 y.o.) had lower bone density. However, this relationship was statistically insignificant (b = −0.068, *t* = −1.18, *p* = 0.24). It is worth pointing out that patients in the age group 60–70 years old have shown positive prediction for the bone density in the lumbar spine (b = 0.102, *t* = 1.94, *p* = 0.053). Furthermore, the two contributors can account for 73% of the variance in the DEXA hip joint, collectively, F (5,317) =9,47, *p* < 0.001. Looking at the unique individual contributions of the predictors, the results show that high atorvastatin dose (b = −0.243, *t* = −4.49, *p* < *0*.*001*), high rosuvastatin dose (b = −1.61, *t* = −2.97, *p* *=* 0.03) negatively predict bone mineral density. The age above 80 years old was shown as a strong predictor of the hip density, however not statistically significant (b = −0.048, *t* = −0.89, *p* = 0.374). Authors acknowledge the trend of reducing bone density with increased age, which demonstrated both in the lumbar spine and the hip (Fig. 4, Fig. 5)

## 4 Discussion

We illustrated that low BMD is overrepresented in females of postmenopausal age on higher doses of statins. There was no correlation between the patient's age and bone mineral density in our study. However, we acknowledge a decrease in T-scores with the patient's age.

Also, it becomes evident that high doses of hydrophilic statins are superior in supporting bone health, as they correlate with higher bone mineral density. Study results show that osteopenia and osteoporosis are prevalent among females on statin treatment compared to the control group.

One of the first studies where females were separated into groups depending on statin dose was performed in 2002, and no significant difference was found. However, there was a small sample size (*N* = 24) (Hsia et al., 2002). Leutner and colleagues who investigated dose-dependency between osteoporosis and statin therapy concluded that low-dose statin treatment with dosages lower or equal to 10 mg of pravastatin, lovastatin, simvastatin and rosuvastatin was related to higher BMD (Leutner et al., 2019).

In our study, participants on high dose atorvastatin and rosuvastatin had statistically lower BMD than women on low dose therapy (in the lumbar spine and the hip). These findings might influence mortality among the female population by reducing the incidence of neck of femur fractures related death.

Our research was done regarding the superiority of one statin agent over another regarding its effect on bone mineral density. Lin et al. stated in their work that high potency statins influence bone structure more than low potency statins (Lin et al., 2018).

The regression analysis has not shown age as a statistically significant factor in our analysis. This can be explained by the homogeneity of the population investigated. Speculations can be made that these results are secondary to surveying women of a similar age living in one region (probable similarities in the vitamin D input, sun exposure). Further inclusion of co-dependent variables in the regression analysis might change this result as the trend towards a decrease in bone mineral density with an increase in age is noted.

To the best of our knowledge, this is the first study showing the pravastatin's superiority within the group. We state that hydrophilic statins, like pravastatin, have a safer profile in terms of their metabolic effects on bone mineral density. This subsequently might reduce pathological and compression fracture in the spine in the elderly female population.

Limitations of the study. An increase in patients' number on pravastatin therapy is required for further research in this field. The authors acknowledge that a limited number of patients on pravastatin therapy might have contributed to the regression analysis results.

This study did not include a separate correlation analysis between bone mineral density, statin therapy and patient's comorbidities (unfortunately, this data was unavailable to the authors). This requires further investigation, as it is found plausible that older females might have more comorbidities, contributing to the findings. Among such comorbidities, liver disease would be of interest to the authors, as the work of Shahrezaee illustrates statistically significant changes in liver function among subjects on atorvastatin that can subsequently contribute to the change in bone mineral density (Shahrezaee et al., 2018). Also, physical activity and nicotine use were not considered during the analysis.

The current study included only females, and we accept that in the male population, this finding might differ. Previously in the literature, it was noted that the positive effects of statins on bone mineral density are less in females (An et al., 2017).

## 5 Conclusion

The study results support the theory that bone mineral density decreases with an increase in a statin dose. Hydrophilic statins, like pravastatin, have a better metabolic profile than lipophilic agents. Overall, we recommend using statin therapy in lower doses where possible to preserve bone mineral density. We postulate that this may decrease the incidence of pathological spinal fractures, neck of femur fractures and decrease fracture-related mortality.

## Funding

No funding was required to perform this study.

## CRediT authorship contribution statement

**Alisa Antonenko:** Conceptualization, Methodology, Writing – original draft. **Aoife Leahy:** Validation, Writing – review & editing. **Mihaly Babenko:** Software, Data curation. **Declan Lyons:** Resources, Supervision.

## Declaration of competing interest

The authors declare that they have no known competing financial interests or personal relationships that could have appeared to influence the work reported in this paper.
